# Interleukin-6 contributes to myocardial damage in pregnant rats with reduced uterine perfusion pressure

**DOI:** 10.1590/1414-431X20186921

**Published:** 2018-06-11

**Authors:** Lan Ding, Chuanming Bai, Ying Liu

**Affiliations:** 1 Department of Obstetrics, Cangzhou Central Hospital, Cangzhou, China; 2 Department of Cardio-Thoracic Surgery, Cangzhou Central Hospital, Cangzhou, China; 3 Department of Obstetrics, Fourth Hospital of Shijiazhuang, Shijiazhuang, China

**Keywords:** Preeclampsia, Myocardial damage, Reduced uterine perfusion pressure, Interleukin-6

## Abstract

Preeclampsia is one of the most frequent and difficult illnesses in pregnancy, which jeopardizes both mother and fetus. There are several diagnostic criteria for preeclampsia. However, the preeclampsia-associated myocardial damage has not been described. In this study, we employed reduced uterine perfusion pressure (RUPP) to generate a rat model of preeclampsia for the evaluation of myocardial damage in late-gestation rats. The expressions of cardiac injury markers were analyzed by immunohistochemistry and ELISA. The arterial pressure and myocardial tissue velocities were also measured. The role of interleukin (IL)-6 in RUPP-associated myocardial damage was further explored. The results showed that RUPP rats had significant myocardial damage, as demonstrated by the high expressions of myoglobin, creatine kinase isoenzyme, cardiac troponin I, and brain natriuretic peptide. In addition, RUPP increased the mean arterial pressure and the early transmitral flow velocity to mitral annulus early diastolic velocity ratio (E/Ea). Furthermore, IL-6 deteriorated these abnormalities, whereas inhibition of IL-6 significantly relieved them. In conclusion, our study demonstrated that RUPP rats displayed myocardial damage in an IL-6-dependent manner.

## Introduction

Pregnancy-induced hypertension, especially preeclampsia, is one of the most frequent and difficult illnesses in pregnancy, which jeopardizes both mother and fetus (1). The International Society for the Study of Hypertension in Pregnancy has provided the following parameters for diagnosing preeclampsia: blood pressure at least 140/90 mmHg on two separate occasions, 4–6 hours apart, coupled to the appearance of protein in the urine corresponding to 300 mg/dL in 24-h collection (2). Preeclampsia is estimated to complicate 2–8% of all pregnancies (3), and it causes maternal morbidity and mortality primarily through cardiovascular complications (4,5). Moreover, preeclampsia has been established as a cardiovascular risk (6). Although preeclampsia usually subsides with the delivery of the placenta, cardiovascular risks associated with hypertension during pregnancy continue in the postpartum period (7). However, the hypertension-induced myocardial damage and its mechanisms are not fully elucidated.

Numerous studies have shown that preeclampsia is associated with chronic immune activation. The increased production of inflammatory cytokines and decreased expression of regulatory and anti-inflammatory cytokines further promote an inflammatory state (2). During preeclampsia, increased levels of tumor necrosis factor (TNF)-α and interleukin (IL)-6 are present in the circulation and in the trophoblast cells of the placenta, whereas the content of IL-10 and IL-4 is decreased (8). This imbalance is associated with placental ischemia and inflammation, which contributes to the exacerbation of preeclampsia (9).

In this study, we explored the myocardial damage in a reduced uterine perfusion pressure (RUPP) rat model. In addition, the role of inflammatory cytokine IL-6 in RUPP-associated myocardial damage was explored.

## Material and Methods

### Reagents

Recombinant rat IL-6 was obtained from PeproTech (USA). Tocilizumab was purchased from Roche Life Science (USA).

### Animal model and experimental drug treatment

Pregnant Sprague-Dawley rats weighing 250–300 g were purchased from the Institute of Zoology, Chinese Academy of Medical Sciences (China). Rats were housed under controlled conditions (25±2°C, 70% humidity and 12-h light-dark periods) and fed a regular sterile chow diet and water *ad libitum*. The experimental protocols were performed according to relevant national and international guidelines and were approved by the Animal Experimental Ethical Committee of Hebei Centers for Disease Control and Prevention.

Thirty rats were randomly divided into the following five groups: 1) pregnant control; 2) sham operation; 3) RUPP operation; 4) RUPP plus IL-6; and 5) RUPP plus tocilizumab. At day 14 of gestation, all rats in the RUPP groups were clipped as previously described (10). IL-6 (50 ng/day) and tocilizumab (8 mg/kg) were administrated to rats intraperitoneally. The mean arterial pressure (MAP) and velocities were measured on day 19 followed by collection of blood and tissue samples.

### Enzyme-linked immunosorbent assay (ELISA)

The expressions of IL-6, cardiac troponin I (cTnI), creatine kinase isoenzyme (CK-MB), myoglobin (Mb), and brain natriuretic peptide (BNP) in serum were detected by ELISA assay according to the manufacturer’s instructions (Sigma, USA). Briefly, serum samples were precoated onto ELISA plates and served as the antigen. o-Dianisidine was used as substrate and the absorbance of the colored horseradish peroxidase product was measured spectrophotometrically at 405 nm by an automated microplate reader (ThermoFisher Scientific, USA).

### Immunohistochemistry

The expressions of IL-6 and BNP in cardiac tissues were assayed by immunohistochemistry. Myocardium sections were incubated with IL-6 or BNP antibody (Cell Signaling Technology, USA) overnight at 4°C, followed by incubation with fluorophore-conjugated secondary antibody (Invitrogen, USA) for 1 h at 4°C. Sections were visualized with a fluorescent microscope (Leica, Germany).

### Measurement of arterial pressure and myocardial tissue velocities

Arterial pressure was monitored with a pressure transducer connected to an arterial pressure-recording device. The myocardial systolic peak velocity (Sm), early diastolic (Ea), and late diastolic velocities (Aa) in three cardiac cycles were measured using tissue Doppler echocardiography (Acuson Sequoia, USA) (11,12). Three-millimeter Doppler sample volume was applied parallel to the right ventricle free wall at the tricuspid valvular level to avoid sampling the right heart cavities due to normal cardiac movement.

### Statistical analysis

Data are reported as means±SD. The difference between groups was analyzed using one-way ANOVA and was considered statistically significant at P<0.05.

## Results

### IL-6 expression in RUPP-induced gestational hypertension rats

**Figure 1.**
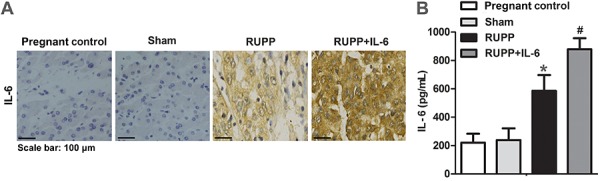
Rats were subjected to reduced uterine perfusion pressure (RUPP) or sham operation and then intraperitoneally injected with interleukin (IL)-6 (50 ng/day) for 5 days. *A*, Expression of IL-6 in cardiac tissue was assayed by immunohistochemistry. Scale bar: 100 μm. *B*, Expression of IL-6 in serum was measured by ELISA. Experiments were repeated at least three times (n=6). Data are reported as means±SD. *P<0.05 *vs* control, ^#^P<0.05 *vs* RUPP (one-way ANOVA).

To gain insights into the function of IL-6 in RUPP-induced gestational hypertension rats, the expression of IL-6 was detected in the myocardial tissue and serum of pregnant rats operated with RUPP. As shown in Figure 1A, the expression of IL-6 was increased in heart tissue after RUPP and was further enhanced by IL-6 treatment. Similar findings were observed in serum (Figure 1B). These results indicated that IL-6 is highly expressed in RUPP-induced gestational hypertension rats.

### IL-6 accelerated RUPP-induced myocardial damage

**Figure 2.**
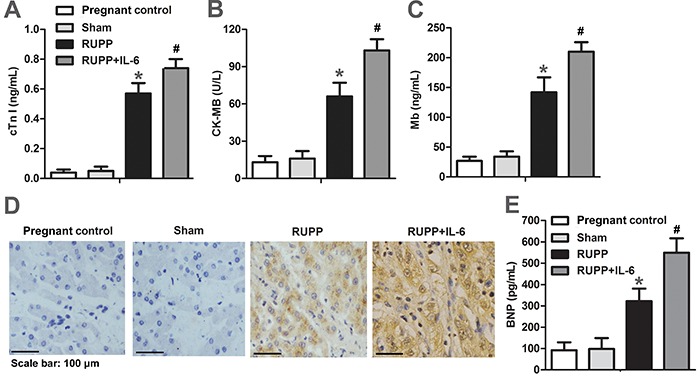
Rats were subjected to reduced uterine perfusion pressure (RUPP) or sham operation and then intraperitoneally injected with interleukin (IL)-6 (50 ng/day) for 5 days. *A*-*C*, Concentrations of cardiac troponin I (cTnI), creatine kinase isoenzyme (CK-MB), and myoglobin (Mb) in serum were detected by ELISA. *D* and *E*, Expressions of brain natriuretic peptide (BNP) in cardiac tissue and serum were detected by immunohistochemistry and ELISA. Scale bar: 100 μm. Experiments were repeated at least three times (n=6). Data are reported as means±SD. *P<0.05 *vs* control, ^#^P<0.05 *vs* RUPP (one-way ANOVA).

To determine the myocardial damage in the RUPP rats, biomarkers of myocardial damage in serum samples were detected by ELISA assay. The levels of cTnI, Mb, and CK-MB in healthy pregnant rats were less than 0.1 ng/mL, less than 72 ng/mL, and 0–24 U/L, respectively. As shown in Figure 2 A-C, the expressions of cTnI, Mb, and CK-MB were markedly increased in RUPP rats and were further enhanced by IL-6 treatment. Since increased level of BNP is one of the key indices of progressive ventricular dilatation, the expression of BNP was detected by immunohistochemistry and ELISA. As shown in Figure 2 D-E, a significant increase of BNP level was detected in RUPP rats. These results indicate that IL-6 may contribute to the myocardial damage in RUPP rats.

### IL-6 promoted RUPP-induced elevation of MAP and E/Ea

**Figure 3.**
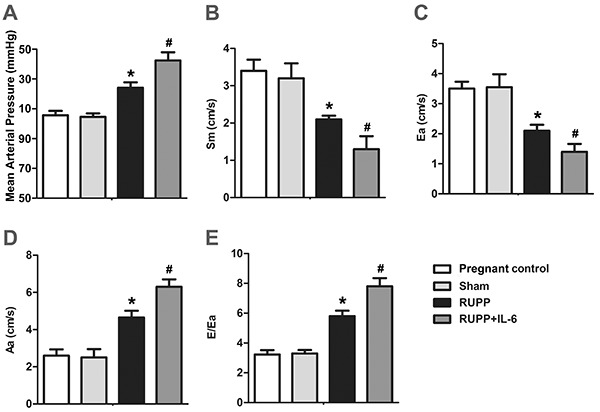
Rats were subjected to reduced uterine perfusion pressure (RUPP) or sham operation and then intraperitoneally injected with interleukin (IL)-6 (50 ng/day) for 5 days. *A,* Arterial pressure was monitored with a pressure transducer connected to an arterial pressure-recording device. *B*-*D*, Myocardial systolic peak velocity (Sm), early diastolic velocity (Ea), and late diastolic velocity (Aa) in three cardiac cycles were measured using tissue Doppler echocardiography. *E*, Early diastolic/late diastolic velocities ratio (E/Ea). All experiments were repeated at least three times (n=6). Data are reported as means±SD. *P<0.05 *vs* control, ^#^P<0.05 *vs* RUPP (one-way ANOVA).

As shown in Figure 3A, the level of MAP was significantly increased after RUPP surgery and was further enhanced by IL-6 treatment. We next examined the Doppler echocardiographic parameters of RUPP hearts. The Sm and Ea peak velocities were significantly decreased in RUPP rat hearts and further reduced by IL-6 treatment, whereas the Aa displayed an opposite result (Figure 3 B-D). E/Ea ratio calculated from medial mitral annulus was also increased with IL-6 (Figure 3E). These results further demonstrated that IL-6 accelerated RUPP-induced myocardial damage.

### Tocilizumab attenuated RUPP-associated myocardial damage

**Figure 4.**
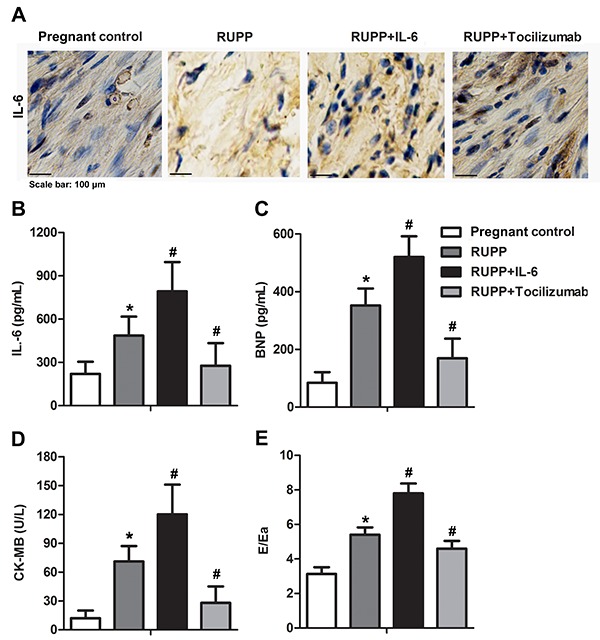
Rats were subjected to reduced uterine perfusion pressure (RUPP) or sham operation and then intraperitoneally injected with interleukin (IL)-6 (50 ng/day) and tocilizumab (8 mg/kg) for 5 days. *A*, Expression of IL-6 in cardiac tissue was assayed by immunohistochemistry. Scale bar: 100 μm. *B*-*D*, Expression of IL-6, brain natriuretic peptide (BNP), and creatine kinase isoenzyme (CK-MB) in serum was measured by ELISA. *E*, Early diastolic/late diastolic velocities ratio (E/Ea). All experiments were repeated at least three times (n=6). Data are reported as means±SD. *P<0.05 *vs* control, ^#^P<0.05 *vs* RUPP (one-way ANOVA).

To further determine whether IL-6 expression is indispensable for the RUPP-induced myocardial damage, tocilizumab, a specific inhibitor of IL-6, was administered to rats after RUPP surgery. As shown in Figure 4A and B, tocilizumab effectively blocked the expression of IL-6 in RUPP rats. Tocilizumab also significantly decreased the expressions of BNP and CK-MB in RUPP hearts (Figure 4C and D). More importantly, the Doppler parameter E/Ea was markedly reduced by tocilizumab administration (Figure 4E). These results strongly indicated that IL-6 was crucial for the progression of myocardial damage in RUPP rats.

## Discussion

There are several diagnostic criteria for preeclampsia, including elevated blood pressure, proteinuria, thrombocytopenia, renal insufficiency, impaired liver function, pulmonary edema, and cerebral or visual symptoms. Furthermore, in the absence of proteinuria, there are many other indicators for preeclampsia, such as doubling serum creatinine or liver transaminases, which indicate renal insufficiency or impaired liver function (13). However, preeclampsia-associated myocardial damage has not been described. In this study, we found that RUPP rats, a commonly used animal model of preeclampsia, have severe myocardial damage, which is attributed to the high expression of IL-6.

Preeclampsia is thought to be caused by a reduction in uterine blood flow due to abnormal trophoblast invasion of the spiral arteries. The development of a model that exploits this reduction in uterine perfusion pressure and flow is a natural alternative for study (10). RUPP models have been performed in many kinds of experimental animals (14–17). The most well-characterized and utilized model is the RUPP pregnant rat model. The RUPP rat mimics numerous physiological features of preeclampsia in women, including hypertension, proteinuria, impaired renal function, and increased vascular reactivity, leptin, and blood lactate (18,19). Fetal intrauterine growth retardation also occurs in RUPP rats, with decreased litter size and pup weight (20,21). Thus, the RUPP rats have many features similar to those of humans with preeclampsia.

Cytosolic enzymes such as CK-MB, LDH, AST, and ALT serve as sensitive indicators to assess the severity of myocardial infarction (22). Increased activities of these enzymes in serum are indicators of cellular damage and loss of functional integrity and/or permeability of cell membrane (23). Myoglobin is a sensitive marker for muscle injury, and its elevated level has specificity for acute myocardial infarction. cTnI is a low molecular weight and contractile protein that is normally not found in serum but is released when myocardial necrosis occurs. Recent studies have indicated that measurement of cTnI may be even more significant in diagnosing acute myocardial injury and for risk prediction in subsequent infarction (24). In the present study, we found that CK-MB, Mb, and cTnI were highly expressed in RUPP rats and further enhanced by IL-6. This observation strongly indicated that RUPP rats were accompanied by myocardial damage.

D’Andrea et al. (12) detected a correlation between myocardial performance assessed at rest via tissue Doppler and cardiac performance during physical effort in adult patients. E/Ea ratio reflects myocardial diastolic properties, and its elevation is an indicator of early heart failure (25). In this study, the E/Ea ratio was increased in RUPP rats and further enhanced by IL-6, indicating that RUPP-induced myocardial damage is closely associated with the level of IL-6.

BNP plays a compensatory role in cardiac disease states due to its diuretic, natriuretic, and vasodilating actions and inhibitory effects on the RAAS and endothelin systems (26). Furthermore, BNP correlates better with the severity of congestive heart failure than atrial natriuretic peptide (27). Therefore, plasma BNP has been suggested to diagnose heart failure (28). In this study, tissue and serum BNP levels were significantly increased after RUPP surgery and IL-6 treatment. Moreover, BNP was significantly reduced by IL-6 inhibitor in RUPP rats. BNP is mainly produced by over-stretched ventricular myocytes (29). Thus, in the current setup, increased BNP levels can represent the direct effect of IL-6 on myocytes.

Inflammatory cytokines such as tumor necrosis factor (TNF)-α are elevated in preeclamptic women and are thought to be an important link between placental ischemia and endothelial dysfunction (10,30). In RUPP rats, the serum level of TNF-α was increased, and infusion of TNF-α increased blood pressure in healthy pregnant rats, suggesting that inflammation could contribute to the hypertension of RUPP rats (31). Supporting this idea, administration of etanercept, a TNF-α soluble receptor, attenuated hypertension, decreased ET-1 transcript expression, and inhibited endothelial cell activation in RUPP rats (32). In this study, we found that IL-6, a target protein of TNF-α, was also elevated in the serum and heart tissues of RUPP rats, and played a crucial role in RUPP-induced myocardial damage.

In conclusion, our study first demonstrated that RUPP rats displayed myocardial damage in an IL-6-dependent manner. However, several limitations of this study should be pointed out, especially the RUPP model. First, hypertension does not always occur in all species with RUPP. Second, RUPP does not mimic all the pathological features of preeclampsia, such as glomerular endotheliosis and changes in hemoglobin, platelets, and liver function (10). Despite these limitations, RUPP model shares the pathogenesis with preeclampsia to a certain extent. Thus, our finding proposed a possible mechanism for preeclampsia-associated myocardial damage.
